# Elevated preoperative platelet distribution width predicts poor prognosis in Esophageal Squamous Cell Carcinoma

**DOI:** 10.1038/s41598-019-51675-y

**Published:** 2019-10-23

**Authors:** Qian Song, Jun-zhou Wu, Sheng Wang, Wen-hu Chen

**Affiliations:** 1 Institute of Cancer and Basic Medicine (ICBM), Chinese Academy of Sciences; Department of Clinical Laboratory, Cancer Hospital of the University of Chinese Academy of Sciences; Department of Clinical Laboratory, Zhejiang Cancer Hospital, Hangzhou, Zhejiang People’s Republic of China; 2 Institute of Cancer and Basic Medicine (ICBM), Chinese Academy of Sciences; Cancer Research Institute, Cancer Hospital of the University of Chinese Academy of Sciences; Cancer Research Institute, Zhejiang Cancer Hospital, Hangzhou, Zhejiang People’s Republic of China

**Keywords:** Prognostic markers, Oesophageal cancer, Tumour biomarkers

## Abstract

Activated platelets play a multifaceted role in tumorigenesis and progression. Platelet distribution width (PDW) is generally applied platelet parameters from routine blood test. Preoperative PDW has been considered a prognostic factor in many cancers. Nevertheless, the prognostic value of PDW in esophageal squamous cell carcinoma (ESCC) remains unknown. The study aimed to investigate whether preoperative PDW could serve as a prognostic factor in patients with ESCC. A total of 495 patients with ESCC undergoing curative surgery were enrolled. The relationship between PDW and clinical features in ESCC was analyzed using chi-square tests. Receiver operating characteristic (ROC) curve was used to determine the optimal cut-off value. Overall survival (OS) and disease-free survival (DFS) stratified by PDW were evaluated by Kaplan–Meier method and log-rank test. Univariate and multivariate Cox regression were used to evaluate the prognostic effect of PDW. Of the 495 patients, elevated PDW was observed in 241(48.7%) of the patients, respectively. An elevated PDW was correlated with depth of tumor (T stage, P = 0.031), nerve infiltration (P = 0.016), hospital time after operation (P = 0.020), platelet (P < 0.001), red cell distribution width (P < 0.001), and aspartate transaminase (P = 0.001). Moreover, elevated PDW (PDW ≥ 13.4 fL) predicted a worse OS and DFS in patients with ESCC (both P < 0.001). Multivariate analyses revealed that PDW was independently associated with OS (hazard ratios 1.194; 95% confidence interval 1.120–1.273; P < 0.001) and DFS (hazard ratios 2.562; 95% confidence interval 1.733–3.786; P < 0.001). Our findings indicated that elevated PDW could serve as an independent worse survival in ESCC.

## Introduction

Esophageal cancer is the sixth and fourth cause of cancer-related mortality in the world and in China^1,2^, with ESCC accounting for 90% of all diagnosed esophageal cancer cases^3^. Although much progress has been achieved in the diagnosis and treatment, the prognosis of ESCC still remains unfavorable^4–6^. Currently, several factors are related to the outcome of ESCC including TNM stage and tumor differentiation. Nevertheless, even within the same staging category, there is disparate prognosis of ESCC because TNM stage could not reflect biological heterogeneity^7^. Therefore, identification of new and accurate prognosis biomarkers in patients with ESCC is of great importance. A growing number of studies have suggested that platelets play a vital role in tumor development, progression and metastasis^8,9^. Platelets take part in the different steps of angiogenesis including proliferation, migration, extracellular matrix degradation, and adhesion of endothelial cells^10^. Activated platelets are involved at cancer-associated thrombosis by releasing inflammatory information, and interacting with neutrophils and monocytes. In addition to activated platelets, an elevated platelet count that has been found in cancer patients seem to be related to a higher proportion of cancer-related venous thromboembolism^11^. Due to these mechanisms, platelets may serve as a potential therapeutic target^12^. Some platelet indices including the platelet count (PLT), platelet distribution width (PDW), and platelet-lymphocyte ratio (PLR), can be readily available and have been confirmed to be associated with the prognosis of various cancers, such as non-small cell lung cancer, pancreatic adenocarcinoma, cervical cancer, and colon cancer^13–17^.

Recently, some researches have showed that an increased pretreatment PLT or PLR could serve as an independent prognosis factor in patients with ESCC^18,19^. However, whether PDW is related to the prognosis in ESCC remains unknown. Therefore, the aim of this retrospective study was to evaluate the prognostic value of PDW in ESCC, and to investigate the relationship between PDW and the clinical-pathological features.

## Results

### Patient characteristics

After screening, 495 patients (428 male and 67 female) with complete follow-up data were enrolled in the final study. The median age at diagnosis was 62 years (Interquartile range: 55–67 years). 38 (7.8%) with well differentiated pathology grade, 326 (67.1%) with middle differentiated pathology grade, 121 (24.9%) with poorly differentiated pathology grade, and 1 (0.02%) with undifferentiated pathology grade. In addition, 223 (45.1%) had high- pathological stage (≥TNM3a-3c), 181 (36.6%) had middle- pathological stage (=TNM2a-2b), 91 (18.4%) early- pathological stage (=TNM1a-1b). 264 (53.3%) had lymph node invasion, 138 (27.9%) had vessel invasive, 169 (34.1%) had nerve infiltration, and 339 (68.5%) only received surgery. The median of hospital time after operation was 11(Interquartile range: 10–13), and the median of the PDW was 13.2(Interquartile range: 11.7–15.0). The clinical-pathological features are listed in Table 1.

**Table 1:** Difference in PDW ratio according to clinical characteristics in ESCC patients.

Variables		Cases	
		N	%
Sex	Male	428	86.5
	Female	67	13.5
Age at therapy initiation(years)	Median	62	
	Interquartile range	(55–67)	
Pathology grade	Well differentiated	38	7.8
	middle differentiated	326	67.1
	Poorly differentiated	121	24.9
	Undifferentiated	1	0.02
Depth of tumor	T1a–1b	51	10.3
	T2	100	20.2
	T3	344	69.5
Lymph node metastasis	N0	231	46.7
	N1	165	33.3
	N2	74	14.9
	N3	25	5.1
Pathological stage	1a–1b	91	18.4
	2a–2b	181	36.6
	3a–3c	223	45.1
Vessel invasive	Yes	138	27.9
	No	357	72.1
Nerve infiltration	Yes	169	34.1
	No	326	65.9
Treatment regimen	S	339	68.5
	S plus postoperative C	111	22.4
	S plus postoperative CRT	45	9.1
Hospital time after operation(days)	Median	11	
	Interquartile range	(10–13)	
PDW	Median	13.2	
	Interquartile range	(11.7–15.0)	
Platelet	Median	198.5	
	Interquartile range	(160.0–236.0)	
Albumin	Median	42.1	
	Interquartile range	(39.5–44.2)	
RDW	Median	12.8	
	Interquartile range	(12.3–13.3)	
Aspartate transaminase	Median	22	
	Interquartile range	(19.0–27.0)	
Fibrinogen	Median	3.73	
	Interquartile range	3.19–4.34	
Hemoglobin	Median	13.7	
	Interquartile range	(12.7–14.6)	

Abbreviations: S, surgery; C, chemotherapy; CRT, chemoradiotherapy; PDW, platelet distribution width; RDW, red cell distribution width.

### High PDW is a predictor of adverse pathological features

**Figure 1:**
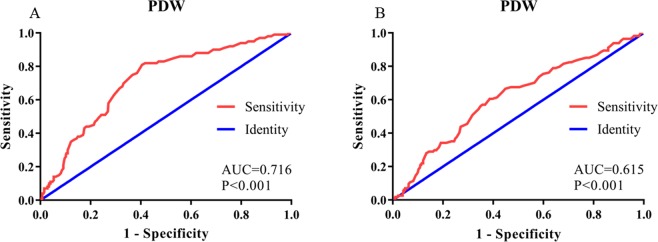
ROC curves analysis of PDW for survival outcomes in patients with ESCC. (**A**) OS revealed the largest AUC (0.716), while PDW cutoff was set at 13.4 for the largest Youden Index (0.405) obtained (sensitivity, 81.0%; specificity, 59.5%). (**B**) DFS revealed the AUC (0.615). OS: overall survival; DFS: disease free survival; PDW: platelet distribution width; AUC: area under the ROC curve; ESCC: esophageal squamous cell carcinoma.

**Figure 2:**
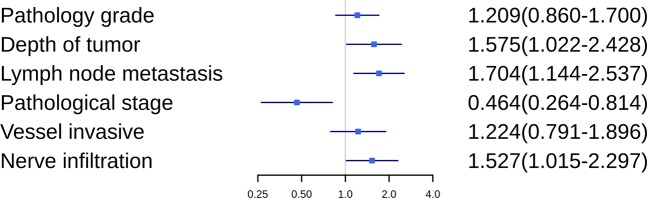
Forest map showing logistic regression analysis of PDW and its predictive value for adverse pathological outcomes.

The areas under the ROC curves (AUCs) were 0.716 and 0.615 for OS and DFS, respectively (Fig. 1). The larger AUC of 0.716 acquired for OS was chose to be the optimal cut-off value of 13.4, with maximum specificity (81.0%) and sensitivity (59.49%) (Fig. 1A). According to the cut-off of PDW, 254 patients (51.3%) with PDW < 13.4 were grouped into the low PDW group, whereas the remaining 241 patients (48.7%) with PDW ≥ 13.4 were divided into the high PDW group. The association between PDW and clinical-pathological features are shown in Table 2. None of the clinical-pathological features was notably related to the PDW including gender, age at diagnosis, pathology grade, lymph node metastasis, pathological stage, vessel invasive, treatment regimen, albumin, fibrinogen, and hemoglobin. However, an elevated PDW was significantly associated with depth of tumor (P = 0.031), nerve infiltration (P = 0.016), hospital time after operation (P = 0.020), platelet (P < 0.001), red cell distribution width (P < 0.001), and aspartate transaminase (P = 0.001). Moreover, high PDW independently predicted depth of tumor (OR = 1.575, P = 0.040), lymph node metastasis (OR = 1.704, P = 0.009), pathological stage (OR = 0.464, P = 0.007), and nerve infiltration (OR = 1.527, P = 0.042) using logistic regression analysis (Table 3 and Fig. 2).

**Table 2:** Relationship between preoperative PDW and clinical-pathological features in patients with ESCC.

Characteristics	Total patients	PDW <13.4 (n = 254)	PDW ≥13.4 (n = 241)	*P* value
Sex	Male	219	209	0.870
	Female	35	32	
Age at therapy initiation(years)	≤60	112	117	0.321
	>60	142	124	
Pathology grade	Well differentiated	22	16	0.390
	middle differentiated	170	156	
	Poorly differentiated	56	65	
	Undifferentiated	0	1	
Depth of tumor	T1a–1b	34	17	**0.031**
	T2	44	56	
	T3	176	168	
Lymph node metastasis	N0	123	108	0.260
	N1	89	76	
	N2	32	42	
	N3	10	15	
Pathological stage	1a–1b	49	42	0.844
	2a–2b	93	88	
	3a–3c	112	111	
Vessel invasive	Yes	63	75	0.117
	No	191	166	
Nerve infiltration	Yes	74	95	**0.016**
	No	180	146	
Treatment regimen	S	163	176	0.102
	S plus postoperative C	64	47	
	S plus postoperative CRT	27	18	
Hospital time after operation(days)	≤14	215	184	**0.020**
	>14	39	57	
Platelet	Median	222.0	171.0	**<0.001**
	Interquartile range	(190.0–257.0)	(142.0–206.0)	
Albumin	Median	42.1	41.9	0.992
	Interquartile range	(39.7–44.1)	(39.3–44.4)	
RDW	Median	12.7	12.9	**<0.001**
	Interquartile range	(12.3–13.2)	(12.4–13.4)	
Aspartate transaminase	Median	21.0	23.0	**0.001**
	Interquartile range	(19.0–26.0)	(19.0–29.0)	
Fibrinogen	Median	3.8	3.7	**0.108**
	Interquartile range	(3.3–4.4)	(3.1–4.3)	
Hemoglobin	Median	13.8	13.7	0.169
	Interquartile range	(12.8–14.7)	(12.6–14.5)	

Abbreviations: S, surgery; C, chemotherapy; CRT, chemoradiotherapy; PDW, platelet distribution width; RDW, red cell distribution width.

**Table 3:** Logistic regression analysis of PDW and its predictive value for adverse pathological outcomes.

Adverse pathological outcomes	Adjusted OR	95% CI	P value
Pathology grade	1.209	0.860–1.7	0.275
Depth of tumor	1.575	1.022–2.428	**0.040**
Lymph node metastasis	1.704	1.144–2.537	**0.009**
Pathological stage	0.464	0.264–0.814	**0.007**
Vessel invasive	1.224	0.791–1.896	0.364
Nerve infiltration	1.527	1.015–2.297	**0.042**

### High PDW is related to poor OS and DFS

**Figure 3:**
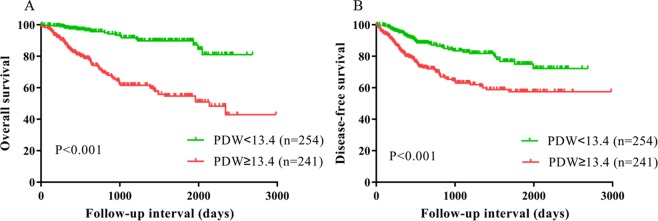
Kaplan–Meier curves for OS (**A**) and DFS (**B**) which was stratified according to PDW value (PDW <13.4 vs. PDW ≥13.4) for ESCC patients after surgery. The difference was evaluated by log-rank tests.

**Figure 4:**
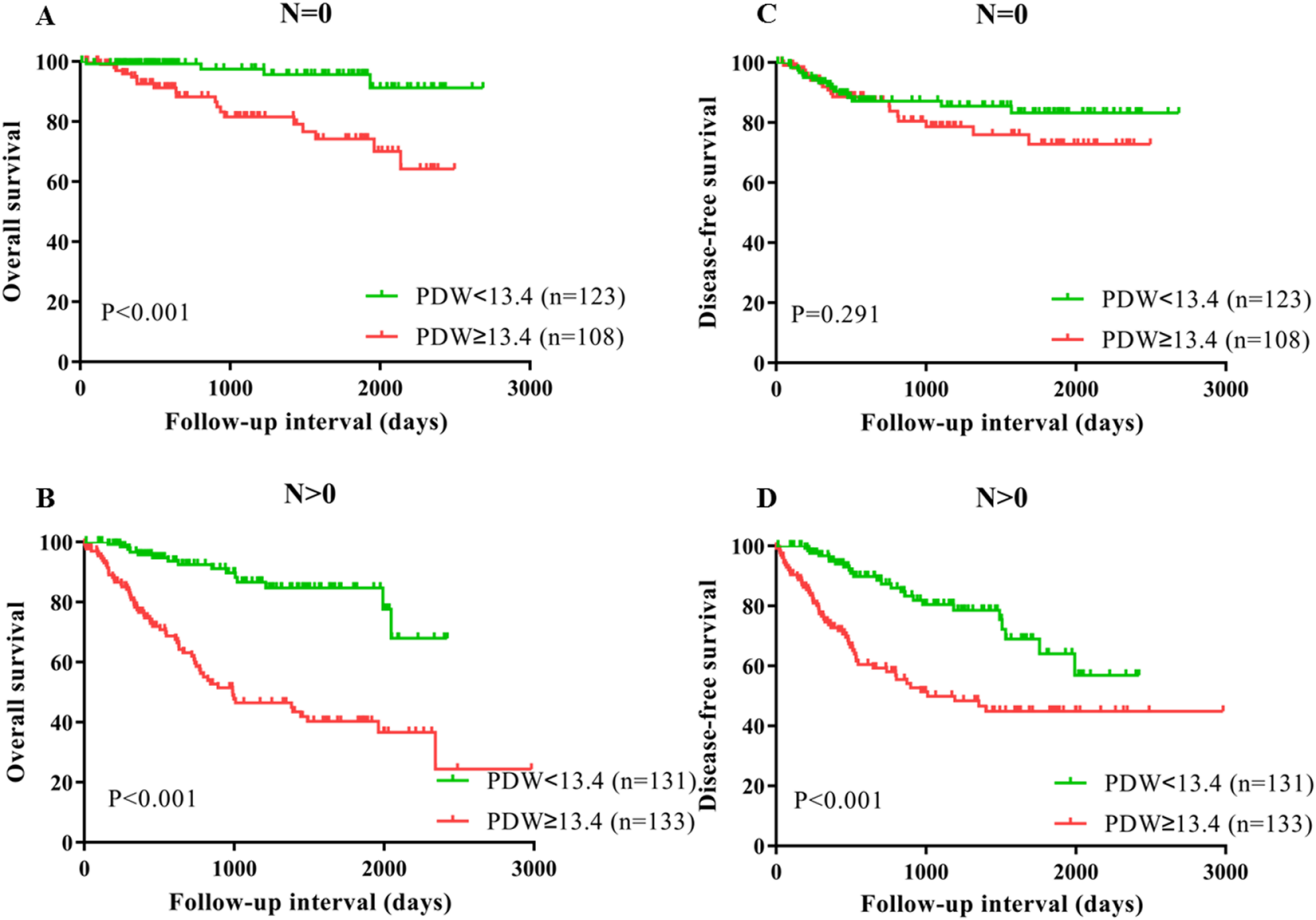
Subgroup analysis based on lymph node metastasis, Kaplan–Meier curves for OS (**A**,**B**) and DFS (**C,D**), which was stratified according to PDW value (PDW <13.4 vs. PDW ≥13.4) for ESCC patients after surgery. The difference was evaluated by log-rank tests.

**Figure 5:**
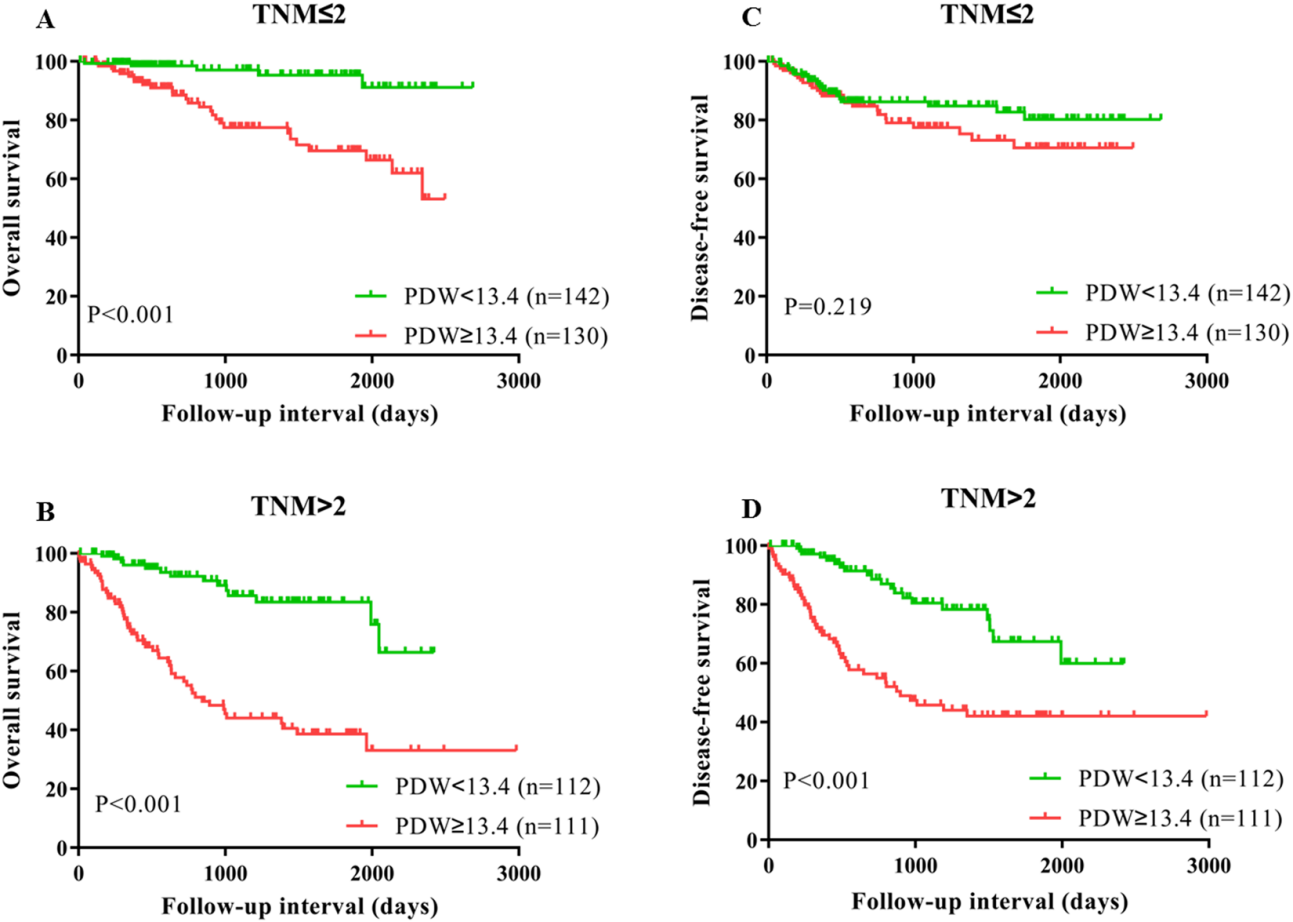
Subgroup analysis based on pathological stage, Kaplan–Meier curves for OS (**A,B**) and DFS (**C,D**), which was stratified according to PDW value (PDW <13.4 vs. PDW ≥13.4) for ESCC patients after surgery. The difference was evaluated by log-rank tests.

The Kaplan–Meier curves exhibited that patients with high PDW had a worse OS (P < 0.001, Fig. 3A) compared with low PDW group. In subgroup analysis according to lymph node metastasis and pathological stage, high PDW was related to worse OS for patients with or without lymph node metastasis (both P < 0.001) and less or more advanced stage (both P < 0.001) (Figs 4 and 5). In addition, univariate analysis shown that high PDW was correlated with worse OS (HR = 5.111, P < 0.001) (Table 4). Using multivariate analysis, high PDW (HR = 1.194, P < 0.001), lymph node metastasis (P < 0.05), nerve infiltration (P = 0.004), and hospital time (P = 0.009) were notable related to worse OS (Table 4).

**Table 4:** Overall survival analyses according to preoperative PDW in 495 patients with ESCC.

Variables	Univariate			Multivariate		
	HR	95% CI	*P* value	HR	95% CI	*P* value
PDW (≥13.4 vs. <13.4)	5.111	3.101–8.425	**<0.001**	1.194	1.120–1.273	**<0.001**
Sex (male vs.female)	1.676	0.845–3.326	0.139			
Age (>60 vs. ≤60)	1.238	0.833–1.838	0.291			
**Depth of tumor**						
T1a–1b	0.296	0.093–0.937	**0.038**	0.447	0.116–1.722	0.242
T2	0.607	0.355–1.038	0.607	0.435	0.135–1.399	0.162
T3	1.000			1.000		
**Lymph node metastasis**						
N0	0.112	0.056–0.222	**<0.001**	0.073	0.015–0.363	**0.001**
N1	0.308	0.164–0.576	**<0.001**	0.331	0.168–0.650	**0.001**
N2	0.432	0.219–0.855	**0.016**	0.486	0.240–0.985	**0.045**
N3	1.000			1.000		
**Pathological stage**						
1a–1b	0.194	0.084–0.447	**<0.001**	2.384	0.184–30.799	0.506
2a–2b	0.395	0.251–0.623	**<0.001**	1.556	0.386–6.283	0.534
3a–3c	1.000			1.000		
Vessel invasive (absence vs. presence)	1.793	1.197–2.686	**0.005**	1.098	0.704–1.713	0.681
Nerve infiltration (absence vs. presence)	1.990	1.343–2.948	**0.001**	1.855	1.214–2.836	**0.004**
**Treatment regimen**						
S	1.425	0.656–3.099	0.371			
S plus postoperative C	1.430	0.611–3.348	0.410			
S plus postoperative CRT	1.000					
Hospital time (days) (>14 vs. ≤14)	1.811	1.169–2.803	**0.008**	1.828	1.159–2.881	**0.009**
Platelet	0.996	0.992–0.999	**0.018**	1.000	0.996–1.004	0.904
Albumin	0.931	0.884–0.981	**0.007**	0.947	0.892–1.006	0.076
RDW	1.258	1.016–1.557	**0.035**	1.072	0.838–1.370	0.579
Aspartate transaminase	0.995	0.972–1.019	0.709			
Fibrinogen	1.137	0.909–1.422	0.262			
Hemoglobin	0.831	0.729–0.948	**0.006**	0.853	0.726–1.002	0.053

Abbreviations: S, surgery; C, chemotherapy; CRT, chemoradiotherapy; PDW, platelet distribution width; RDW, red cell distribution width.

By Kaplan–Meier analysis, the DFS was poor in the high PDW group (P < 0.001, Fig. 3B). Similarly, based on subgroup analysis, with lymph node metastasis (P < 0.001) and advanced stage (P < 0.001) could serve as predictors for short DFS in patients with ESCC, which was not observed in patients without lymph node metastasis (P = 0.291) and less advanced stage (P = 0.219) (Figs 4 and 5). In the univariate analysis, high PDW was a significant predictor of unfavorable DFS (HR = 2.302, P < 0.001) (Table 5). After adjustment for confounders, high PDW (HR = 2.562, P < 0.001), lymph node metastasis (P < 0.05), and surgery (P = 0.047) were correlated with decreased DFS (Table 5). In a word, PDW was an independent prognostic factor for patients with ESCC undergoing surgery.

**Table 5:** Disease-free survival analyses according to preoperative PDW in 495 patients with ESCC.

Variables	Univariate			Multivariate		
	HR	95% CI	*P* value	HR	95% CI	*P* value
PDW (≥13.4 vs. <13.4)	2.302	1.567–3.383	**<0.001**	2.562	1.733–3.786	**<0.001**
Sex (male vs.female)	1.545	0.830–2.878	0.170			
Age (>60 vs. ≤60)	0.881	0.610–1.273	0.501			
**Depth of tumor**						
T1a–1b	0.838	0.435–1.614	0.597			
T2	0.601	0.357–1.011	0.055			
T3	1.000					
**Lymph node metastasis**						
N0	0.160	0.084–0.303	**<0.001**	0.205	0.074–0.569	**0.002**
N1	0.266	0.141–0.500	**<0.001**	0.265	0.136–0.515	**<0.001**
N2	0.471	0.243–0.915	**0.026**	0.424	0.217–0.827	**0.012**
N3	1.000			1.000		
**Pathological stage**						
1a–1b	0.376	0.203–0.694	**0.002**	1.039	0.363–2.975	0.943
2a–2b	0.511	0.337–0.775	**0.002**	1.082	0.517–2.261	0.835
3a–3c	1.000			1.000		
Vessel invasive (absence vs. presence)	1.376	0.927–2.043	0.114			
Nerve infiltration (absence vs. presence)	1.640	1.131–2.380	**0.009**	1.424	0.960–2.113	0.079
**Treatment regimen**						
S	0.496	0.280–0.878	**0.016**	0.551	0.306–0.993	**0.047**
S plus postoperative C	1.344	0.748–2.416	0.323	1.304	0.719–2.364	0.382
S plus postoperative CRT	1.000					
Hospital time (days) (>14 vs. ≤14)	1.214	0.773–1.905	0.399			
Platelet	0.998	0.995–1.001	0.285			
Albumin	0.969	0.922–1.019	0.217			
RDW	1.149	0.931–1.418	0.195			
Aspartate transaminase	0.997	0.975–1.019	0.791			
Fibrinogen	0.931	0.748–1.159	0.524			
Hemoglobin	0.962	0.847–1.091	0.545			

Abbreviations: S, surgery; C, chemotherapy; CRT, chemoradiotherapy; PDW, platelet distribution width; RDW, red cell distribution width.

## Discussion

Numerous researches showed that platelet activation play an important part in cancer progression. Thrombocytosis is related to worse clinical outcome in patients with various cancers, including ovarian cancer, colorectal cancer, and pancreatic cancer^20–22^. The PDW that is one of the platelet indices not merely check platelet volume heterogeneity, but also reactive platelet activity. Recently, several studies revealed that a high PDW is an unfavorable prognosis factor in melanoma patients, laryngeal cancer, and gastric cancer^23–25^. To the best of our knowledge, the prognostic value of the preoperative PDW in ESCC patients remains unknown.

This was the first retrospective research revealed that a PDW with a cut-off 13.4 fL was an independent prognostic factor for the OS and DFS in ESCC patients. Our findings reported that an elevated PDW was correlated with depth of tumor, nerve infiltration, and hospital time after operation. Moreover, high PDW was an independent predictor for ESCC patients with lymph node metastasis according to further subgroup analyses.

Nevertheless, the potential mechanism by which PDW have an effect on cancer progression is unclear. One possible cause is that platelets facilitate the hypercoagulability in tumor. Activated platelets produce a procoagulant micro-environment and aggregate with tumor cell. Platelet-derived growth factor (PDGF) family members including PDGF-A, PDGF-B, PDGF-C and PDGF-D, play a vital role in cancer cell proliferation, apoptosis, transformation, invasion, metastasis and angiogenesis^26–31^. In esophageal cancer, PDGF-D expression is associated with clinical-pathological features and worse survival. Moreover, platelet-derived growth factor-D contributes to proliferation and invasion of esophageal squamous cell carcinoma by up-regulating NF-κB signaling pathways^32^. Consistent with previous studies, our findings indirectly suggested anti-platelet could serve as one part of cancer adjuvant therapy^33^.

Another possible mechanism is that bone marrow cells malfunction may be associated with the lower PDW. PDW reflects platelet heterogeneity, which is caused by heterogeneous demarcation of megakaryocytes^34^. Cytokines, including interleukin-6 (IL-6), macrophage colony stimulating factor (M-CSF), and granulocytes colony stimulating factor (G-CSF), have an effect on megakaryocytic maturation, platelet production, and platelet size^35^. IL-6 facilitates cancer cell proliferation, invasion, and metastasis. IL-6 is correlated with the prognosis and depression of cancer patients and is considered to the therapy target^36–38^. Moreover, G-CSF stimulates megakaryopoiesis and constrains tumor to proliferation. M-CSF was an important factor in the cancer microenvironment, involving in the interactions between tumor-infiltrated macrophages and tumor cells^39–41^. Those reports are in accord with the point that activated platelets participate in the pathogenesis of esophageal cancer.

There were several limitations of our study: first, this was the single-center design and retrospective study, which might have selection bias. Second, the biological mechanism of PDW affecting prognosis need to explored. Third, a controversial cut-off value determined by different ways, such as mean, ROC curve, and C index, could be the optimal predictor of clinical outcome in ESCC patients. In this study, we chose ROC curve to determine the cut-off value. Future studies with multi-center design and prospective trials are necessary to validate the prognostic value of PDW in ESCC patients.

An elevated preoperative PDW indicates a worse OS and DFS of patients with newly diagnosed ESCC undergoing surgery. Our finding may contribute to assess the prognosis of ESCC.

## Methods

### Patient recruitment and data collection

This retrospective study was approved by the Ethics Committee of Zhejiang Cancer Hospital, and included 590 ESCC patients who were newly diagnosed between 2008 and 2013. 95 patients who met the following standard were excluded from the study: neoadjuvant chemotherapy or radiotherapy before surgery; loss to follow-up; data missing; concomitant disease that could interfere with platelet, including autoimmune disease, splenic disease, severe hypertension, and a history of blood transfusion; other factors that could affect the PDW, including megaloblastic anemia, acute myeloid leukemia, splenectomy, giant platelet syndrome, and thrombotic disease. The enrolled 495 patients completed written informed consent.

The pretreatment peripheral blood cell count was checked via a SYSMEX XE-2100 (Sysmex, Kobe, Japan) Automatic Blood Cell Analyzer. The PDW measurement is the first time of admission.

### Follow-up strategy

After surgery, patients were followed up every three months for the first year, six months during the second year and 12 months thereafter. Physical examination, blood routine examination, and medical history were achieved conventionally. Bone scans, chest/abdominal CT/MRI, and chest radiography were acquired when in cases of suspicious metastasis or recurrence.

### Statistical analysis

The PDW was analyzed as continuous variables and the clinical-pathological features were counted as categorical variables. The optimal cut-off value of PDW for predicting survival was determined by the ROC curve analysis. The relationship between PDW and clinical-pathological features in ESCC was analyzed by chi-square tests. The Kaplan-Meier method and the log-rank test were used for the overall survival (OS) and disease-free survival (DFS) analyses. The association between PDW and clinical-pathological features were investigated by logistic regression analysis. Clinical-pathological features with P < 0.01 were selected to be the subgroup factor. Subgroup analysis was based on lymph node metastasis and pathological stage. Whether the OS and DFS was an independent prognosis factor was determined by Cox proportional hazards regression models. Risk factors with P < 0.01 in univariate analysis were chosen to multivariate analyses. The SPSS software version 19.0 (IBM SPSS, Chicago, IL, USA) was utilized for statistical analysis.

### Ethics approval and consent to participate

All procedures in the present study were performed in accordance with the ethical standards of the World Medical Association Declaration of Helsinki. The study approval was obtained from ethics committee at Zhejiang Cancer Hospital and informed consents were informed from all participants.

## Data Availability

The data and materials can be found from the first author and corresponding author.
